# Medical students from German-speaking countries on abroad electives in Africa: destinations, motivations, trends and ethical dilemmas

**DOI:** 10.1186/s12960-022-00707-2

**Published:** 2022-01-17

**Authors:** Maximilian Andreas Storz, Ann-Kathrin Lederer, Eric Pieter Heymann

**Affiliations:** 1 Department of Internal Medicine II, Centre for Complementary Medicine, Faculty of Medicine, University of Freiburg, Freiburg, Germany; 2 Department of Emergency Medicine, Cantonal Hospital of Neuchâtel, Neuchâtel, Switzerland

**Keywords:** Overseas elective, Medical elective, International elective, Travel medicine, Global health, Medical education, Medical ethics, Africa, SARS-CoV-2, Surgery

## Abstract

**Background:**

International medical electives are one the highlights of medical training. Literature about international electives is scarce, and understanding what made a student choose one destination over another is unclear. Many medical students based in Europe travel to Africa each year for their elective, however, students’ expectations and motivations are yet largely unexplored.

**Methods:**

To gain insights into the factors driving students to travel to Africa, we analyzed two large international elective databases based in Germany. We reviewed elective testimonies and extrapolated geographical data as well as the choice of discipline for electives completed in Africa. Based on pre-defined categories, we also investigated students’ motivations and expectations.

**Results:**

We identified approximately 300 elective reports from medical students from German-speaking countries who chose to travel to Africa for their elective. Students commonly reported destinations in Southern and East Africa, with the Republic of South Africa and Tanzania being the most frequently selected destinations. Surgical disciplines were the most commonly reported choice. Diverse motivations were identified, including the desire to improve knowledge and clinical examination skills. A large proportion of students reported a link between destination choice and the potential to partake in surgical procedures not feasible at home; whether these surgeries were not or no longer practiced at home, or whether students could not partake due to level of training, was not ascertainable from the data. A trend-analysis revealed a growing interest in travelling to Africa for electives within the last 15 years. We observed a sharp decline in reports in 2020, a phenomenon most likely related to SARS-CoV-2-related travel restrictions.

**Conclusions:**

This study suggests that medical electives in Africa are commonly reported by medical students from German-speaking countries, with diverse motivations for the choice of destination. A non-neglectable proportion of students identified the possibility to engage in surgical procedures as one of the main reasons for choosing Africa. This poses a series of ethical dilemmas, and well-structured pre-departure trainings may be a solution to this. The recent dip in overseas electives should be seen as a unique opportunity for medical schools and universities to restructure their international elective programs.

## Background

Each year, thousands of medical students undertake foreign clinical electives. Abroad electives are often considered a highlight of medical school experience and usually last between 1 and 4 months [1, 2]. These training opportunities give students the chance to discover a new clinical environment in which medicine is practiced, and learn the art of medicine from a variety of dedicated healthcare professionals. Despite high travel expenses, health and (occasionally) security risks, electives in Africa are traditionally popular [3, 4], with the Republic of South Africa (RSA), Ghana, and Tanzania highly sought-after destinations [5].

Several studies have emphasized the potential (international) medical electives have to improve the participants’ knowledge, skills, and attitudes [6], with students often citing the desire to improve their clinical examination skills and the opportunity to travel and to gain insights into a different cultural and clinical climate as motivation for choosing foreign destinations [1, 6]. Moreover, in light of improved clinical examination skills [7], students travelling abroad for electives in low- and middle-income countries generally reported less dependency on technology as well as improved communication skills and a better understanding of infectious and tropical diseases [8–12].

While international electives may go along with a number of perceived benefits, there are also perceived and potential hazards that warrant further investigation. In general, students travelling abroad for international electives were found to be more frequently exposed to infectious diseases, trauma and other physical injuries as well as psychological distress, and (depending on the elective destination) excessive sun exposure [13], than those who stayed in their home country. Some students also reported violence and other events related to the political situation of an elected country [14].

This appears to be important, as sub-state terrorism endangers the state of peace and security in some African regions [15, 16]. Ongoing security challenges, including police corruption [17], political coups against governments [18] and revolutionary warfare [19] are factors that warrant general consideration when planning an abroad elective. In addition to these potential uncontrollable dangers, other factors such as tropical and infectious diseases [20] and the ongoing COVID-19 pandemic may warrant further reflection [21].

Despite all the aforementioned reported and perceived safety risks and hazards in some regions and countries, it appears that demand for electives from Germany-based students to be based in Africa has been continuously growing. With literature about electives generally scarce [13, 22] and electives being one of the least understood areas of undergraduate medical education [23], we sought to determine what motivations and expectations students have when organizing their time in Africa. We reviewed two large German elective databases to gain insights into students’ preferred destinations and clinical disciplines chosen for an elective in Africa, in a bid to identify potential trends and patterns, as an aid to medical schools in preparing students when going abroad.

## Methods

We interrogated two large German databases cataloguing medical elective testimonies, focusing both on mandatory short-term electives (referred to as Famulatur [from the Latin “famulus”, which translates to “servant”] and long-term final-year electives (known as Praktisches Jahr, German for “practical year”) [24–27]. Both databases, Famulaturranking (www.famulaturranking.de) and PJ-Ranking (www.pj-ranking.de), are in German language and mainly used by German, Austrian and Swiss students [28]. Reports from students across all 3 countries were included in this analysis. The two databases were reviewed and data pertaining to either form of elective having taken place in Africa over a period of 15 years was extrapolated data to a Microsoft Excel File. Excluding duplicate reports, descriptive categories were analyzed using PSPP statistical software ([Version 0.8.5] Free Software Foundation, Boston). We based ourselves on the United Nations Development Program country list [29] for a full list of African nations (e.g., the island of Reunion is thus considered an African nation). Since all reports were anonymous, we could not gather any information on the students themselves (e.g., demographic data or prior abroad elective experience).

In addition, each identified report was reviewed to gain insights into students’ motivations and expectations for an elective in Africa. Based on prior analysis of 50 randomly selected elective reports, we created 10 categories summarizing the most frequently mentioned reasons for an elective in an African country. These categories included: “improved clinical examination skills and expansion of clinical knowledge”, “better understanding of tropical and infectious diseases”, “observation of practice and organization of health care in another country”, “improved communication and language skills”, “working with underserved populations”, “building a personal or professional network”, “experiencing a new culture”, “travel and holiday”, and finally “engaging in surgical procedures and operations not possible at home”. We also added a last category entitled “miscellaneous” for motivations not covered by the other pre-defined categories. Whenever a student expressed his motivation, we tagged the report and assigned it to one of the aforementioned categories. Multiple answers were possible. We considered all reports regardless of their length and language (German and English). Moreover, we included both statements with regard to motivations (“I went to Ghana to learn more about infectious diseases that are uncommon in Europe”) and expectations (“Based on previous reports of electives in urban South Africa, I expected to see a lot of penetrating traumas and firearm injuries”).

We performed the analysis during the first three weeks of March 2021 and applied no time restriction. For interested readers, the general methodology is described elsewhere in detail [4, 28].

## Results

We identified 296 elective testimonies published between 2006 and 2020 reporting an international elective in Africa. 223 reports were uploaded to “PJ-ranking” and 73 elective reports were uploaded to “Famulaturranking”, respectively.

### Destinations

Students travelled to 22 different African countries, including Benin, Cameroon, Ethiopia, Gambia, Ghana, Ivory Coast, Kenia, Malawi, Morocco, Namibia, the Republic of Botswana, the Republic of Mauritius, the Republic of Senegal, the Republic of Seychelles, Réunion, Rwanda, the Republic of South Africa, Tanzania, the Togolese Republic, Tunisia, Uganda and Zambia.

**Fig. 1:**
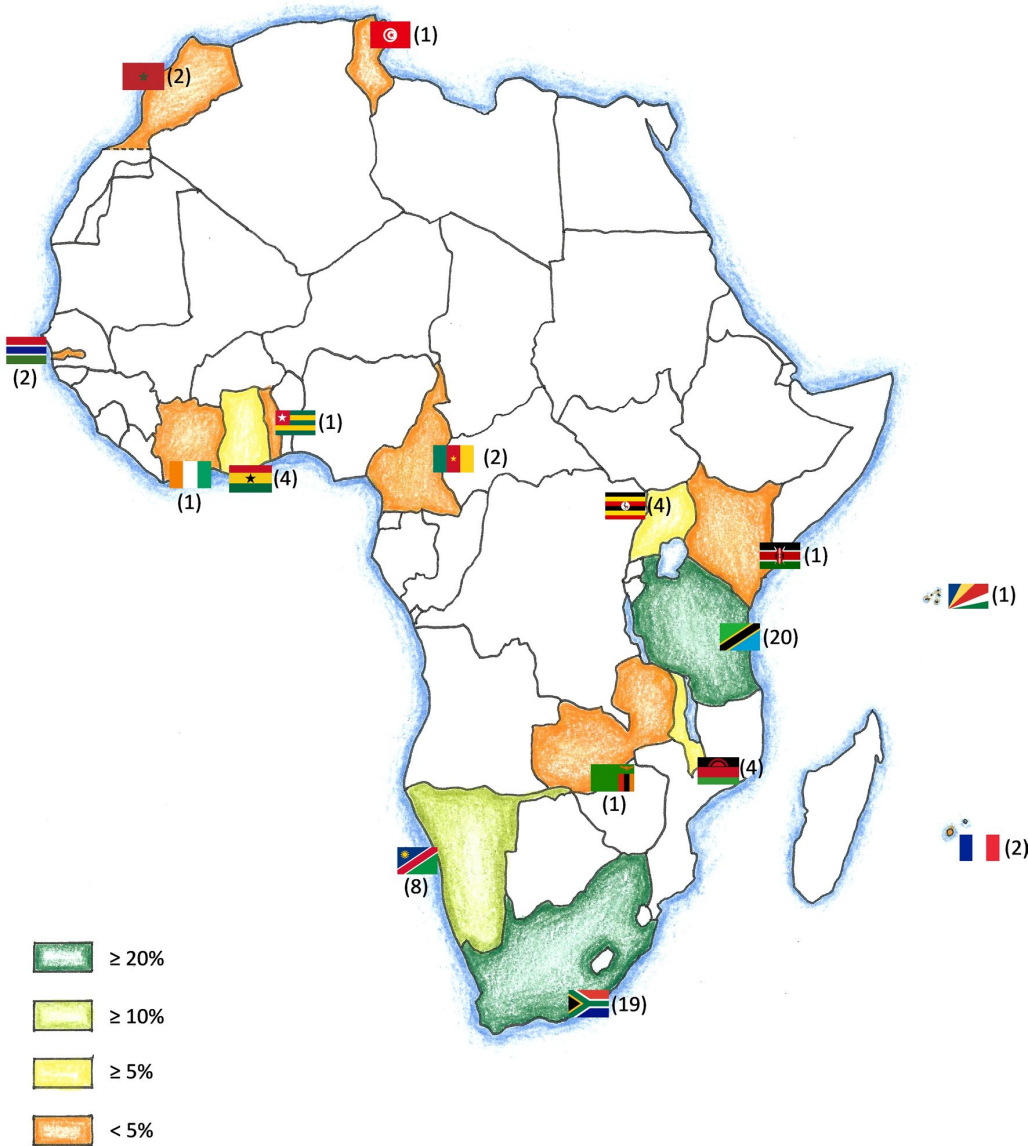
Commonly chosen short-term elective destinations: an overview. The number in parentheses indicates the amount of elective reports available for the respective country

The most frequently chosen destinations for short-term electives were Tanzania (27.40%), South Africa (26.03%) and Namibia (10.96%). Figure 1 shows other short-term elective destinations visited by medical students from German-speaking countries during 2006 and 2020.

**Fig. 2:**
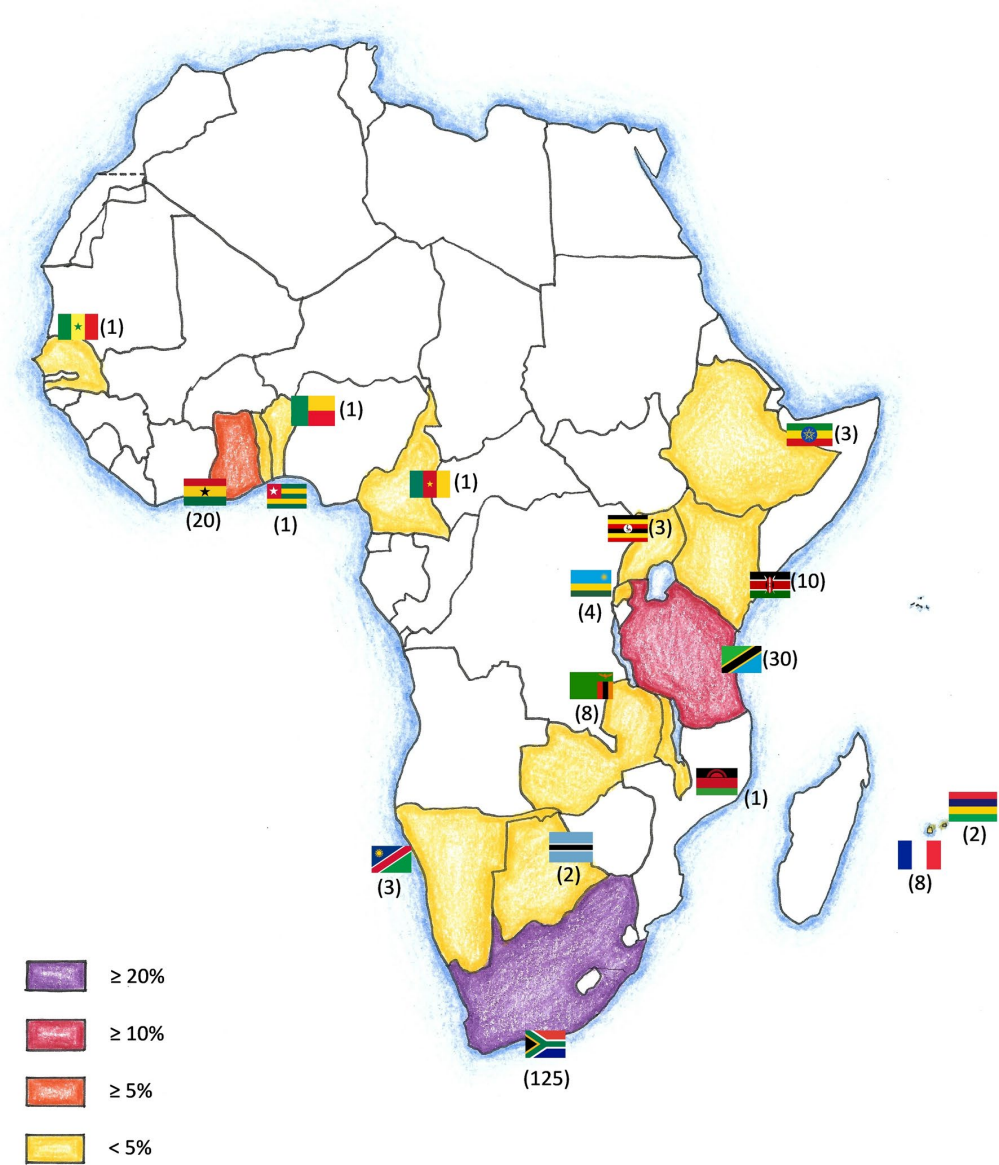
Frequently visited long-term elective destinations: an overview. The number in parentheses indicates the amount of elective reports available for the respective country

With regard to long-term electives, South Africa was the most frequently visited destination (56.05%) in our sample, followed by Tanzania (13.45%) and Ghana (8.97%). Figure 2 shows other frequently chosen destinations for long-term electives and emphasizes large regional differences in terms of popularity. South and East Africa were more often than North and Central Africa.

### Disciplines

Medical students from German-speaking countries reported abroad electives in 25 different clinical disciplines (see Table 1).

**Table 1:** Elective disciplines chosen by medical students from German-speaking countries pursuing an abroad elective in Africa

Clinical discipline	Famulatur *n* =	Practical year *n* =
Anesthesiology	2	2
Cardiology	1	0
Cardiovascular surgery	0	5
Dermatology	0	1
Emergency medicine	7	4
Forensic medicine	0	1
Gastroenterology	0	1
General practice	2	0
General surgery	12	124
Gynecology and obstetrics	14	6
Internal medicine	10	34
Neurology	0	4
Neurosurgery	0	1
Ophthalmology	0	1
Orthopedic surgery	3	3
Oto-Rhino-laryngology	1	0
Pediatric surgery	2	5
Pediatrics	6	6
Plastic surgery	0	7
Pneumology	2	0
Radiology	0	1
Rheumatology	0	1
Trauma surgery	6	15
Tropical medicine	5	0
Vascular surgery	0	1

For short-term electives, Gynecology and Obstetrics was the most commonly selected elective discipline (19.18%) in this sample, followed by General Surgery (16.44%) and Internal Medicine (13.70%). Overall, 27.4% of all short-term elective testimonies reported an elective in a surgical discipline. Tropical medicine-focused electives accounted for approximately 7% of all short-term electives.

For long-term electives, the distribution changed. More than 55% of students performed an elective in General surgery. Other frequently selected subjects included Internal medicine (15.25%) and Trauma Surgery (15%). All surgical disciplines taken together accounted for 72.21% of long-term electives.

### Motivations and expectations

**Fig. 3:**
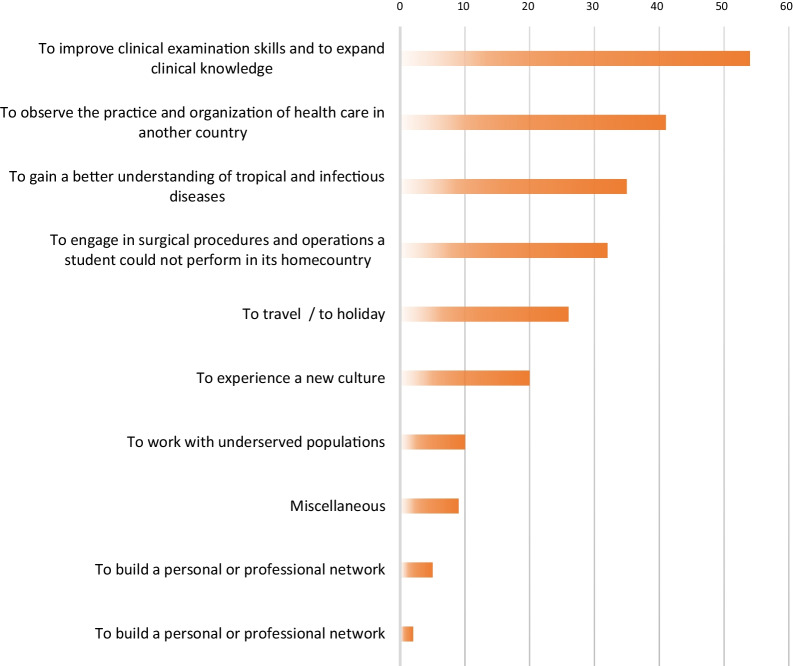
Motivations for an abroad elective in Africa: an overview. N = number of mentions

One hundred students (33.78%) mentioned at least one motivation for (or expectation of) an abroad elective in Africa. Improvement of clinical examination skills and expansion of clinical knowledge were the main reasons for students to go abroad (Fig. 3). Only two students included the potential for building a personal or professional network as a reason for their destination choice, whilst five students mentioned the possibility of improving communication and language skills.

Nine students expressed motives falling in the category “miscellaneous”. One student went to Africa to learn more about a local UNICEF (United Nations International Children’s Emergency Fund) nutrition program and another student hoped to gain better insights into vaccination programs in Africa.

Other motivations included gaining insights into community services and school programs (*n* = 1), learning more about Apartheid, South African history and Nelson Mandela (*n* = 1), and exploring whether development aid could be a suitable career goal (*n* = 1). One student had had positive prior experiences in Africa and two others wanted to learn more about cost-efficient medicine not relying on technical devices.

### Trends

**Fig. 4:**
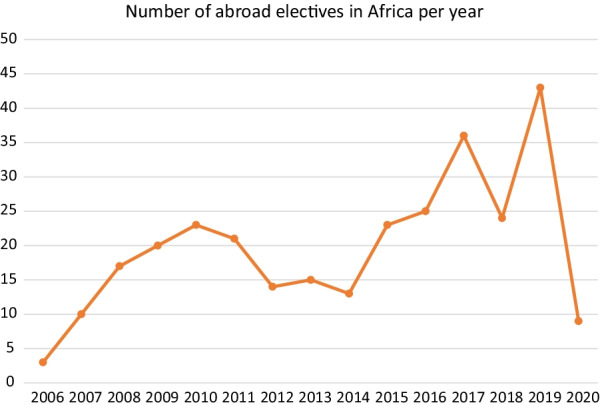
Number of abroad elective reports uploaded to “PJ-ranking” and “Famulaturranking” by medical students from German-speaking countries per year (2006–2020)

Our data suggest that the number of medical students from German-speaking countries who travelled to Africa for their medical elective has increased over the past 15 years, with a clear increase in the number of reports between 2006 and 2010 as well as between 2014 and 2017. The maximum number of reports published was in 2019, followed by a sharp decline in 2020. Figure 4 shows the number of reports published per year (short- and long-term electives combined).

## Discussion

For this study, we analyzed the two largest German online databases for international medical electives. Our in-depth analysis provided many important insights into medical students’ elective preferences and their motivations for an abroad elective in Africa.

We identified approximately 300 elective reports in this sample, demonstrating a clear trend for destinations in Southern Africa and East Africa (Figs. 1 and 2). The Republic of South Africa (RSA) and Tanzania were the most commonly selected destinations, with surgical disciplines being the preferred choice in this sample. In addition, data analysis revealed a diverse range of motivations to travel to Africa. Three main topics, namely ethical considerations, recent trends and safety aspects discussed below.

### Ethical considerations

While many students expressed the wish to improve their clinical examination skills and clinical knowledge, a large proportion mentioned an interest in travelling to Africa to partake in surgical procedures which would otherwise not have been possible in their home countries (Fig. 3). Either from lack of cases or simply because these procedures are no longer done at home, this argument brings with it an ethical dilemma: are electives becoming medical (experience) tourism rather than purely formative training?

As increasing consideration is being given to the ethics of international electives in their current form [30], studies have begun emphasizing the potentially negative consequences for host communities [31]. With international students often paying tuition and administration fees to host facilities, there is a growing expectation of supervision and teaching, which could theoretically result in local staff neglecting their clinical time, in places where staff numbers are already low. This brings forth another point: these electives serve as a teaching opportunity for students, and as such, students require proper supervision and support [32], which could potentially exacerbate the lack of resources and local staff time for both supervision and teaching [30, 33].

This may prompt a debate as to whether electives should be limited to centers where sufficient staff numbers are available, to allow both teaching and training of international candidates, whilst ensuring continuous patient care. Whilst this argument is defendable, it would undoubtedly limit the number of places international electives could be held at, and diminish income to institutions who may otherwise rely on this additional form of revenue (from international student fees). A potential solution would be to limit electives to centers where local in-country medical students train; thus, international students could learn alongside national students in already established local clinical training programs.

Without adequate supervision, students are more frequently placed in situations where they are expected to take on the role of a qualified physician [32, 34]. As such, students are given responsibility for patients [35], and asked to perform potentially dangerous procedures on their own [32, 36]. Although deontologically incorrect, justification for this is based on a utilitarian viewpoint, arguing that no one would look after these patients if elective students were not present [32]. Such behavior can potentially result in morbidity and mortality, and constitutes a serious ethical breach of practicing beyond one’s competency [37, 38]. This appears to be especially true where electives lack a structured plan and/or curriculum [32, 39], and could be used as an argument to limit electives to these sites for only advanced medical students (PJ).

To tackle this problem, many schools have developed global health curricula in an effort to provide ethical training for medical students, often in the form of peripheral pre-departure trainings [40]. Our findings suggest that such pre-departure trainings should explore students’ motivations and provide support for potentially arising conflicts (e.g., when students are expected to take on the role of a qualified doctor). A well-structured elective curriculum, portraying expectations and standards (not just in terms of global health, but also in terms of clinical (examination) skills), is essential, and something that we highly recommend medical schools establish and share with receiving facilities.

In light of the increasing number of medical students from German-speaking countries going to Africa (Fig. 4), it is time to explore their motivations in greater detail. Since the COVID-19 pandemic has resulted in such programs being placed on halt in many areas (see below), it would now be an excellent opportunity to restructure pre-departure trainings, taking students’ elective motivations into account.

### Recent trends

Looking at more recent trends, there has been a sharp decline in overseas electives from 2020. This phenomenon is most likely a consequence of the COVID-19 pandemic and subsequent travel restrictions [28, 41]. In light of the importance electives have for medical students, it is likely that numbers will rise again after the pandemic, with some studies suggesting that students could provide a valuable temporary workforce for struggling health systems during these unprecedented times [42]. We believe that the current paucity in international travel constitutes a great opportunity for medical schools involved in elective planning, as this gives them an opportunity to review (and restructure where needed) their entire system, from medical ethics curricula to elective catalogues, pre-departure trainings and elective center certifications.

### Safety aspects

A final aspect that warrants investigation is health and safety during medical electives. International medical placements tend to be of higher risk than clinical electives at home [43]. From unfamiliar settings to loss of (extrinsic) references, a recent publication by Watson et al. discussed recommendations covering 11 different themes that are likely to improve the safety and quality of an elective program [43]. The authors also emphasized that some medical schools prohibited electives in surgery and obstetrics in some sub-Saharan African regions in the past.

In contrast to this, our data suggest that surgical electives in the RSA were frequently chosen by medical students from German-speaking countries in our sample (Figs. 1 and 2). This country bears the greatest burden of the HIV epidemic [44] and it is therefore of paramount importance to equip students with practical knowledge about this topic before going abroad. This might be especially true with regard to the high amount of electives in surgical disciplines (Table 1). Johnston et al. recently emphasized that medical students may mitigate health risks during an elective by being informed and well prepared for high-risk situations—a fact that might apply particularly for infectious diseases [45].

While further large-scale epidemiological studies are necessary to evaluate the burden of infectious diseases during abroad medical electives [46], it is also important to have a geographical understanding of popular student destinations. This allows for theoretical conclusions about what diseases are to be expected in certain areas and helps students to better prepare for their electives. Our geographical analysis (Figs. 1 and 2) might be of great help in this context and form the basis for country-specific preparation guidelines.

### Strengths and limitations

Our manuscript has several strengths and weaknesses that warrant further investigation. We present a relatively large dataset of approximately 300 elective reports of medical students from German-speaking countries who went to Africa. To the best of our knowledge, a comparably detailed analysis over such a large timeframe (2006–2020) has not been conducted before. Our data investigated students’ motivation for an abroad elective in Africa, an important feature for those involved in the development of abroad elective frameworks and guidelines, and medical education as a whole. Our findings highlight the need for improved pre-departure trainings that also cover moral ethics and social theory training. Moreover, we provide insights into commonly reported elective disciplines which may help universities and other institutions tailor subject-specific recommendations, e.g., for the high number of abroad surgical electives.

Noteworthy, the present analysis has several limitations. Uploading elective reports is not mandatory and students receive no credit for this. Thus, it is likely that the actual number of students going to Africa for an abroad elective is much higher. The fact that these reports are not mandatory is also a potential selection bias, as only motivated students invested the time to write a report. It is not inconceivable that students going abroad to holiday do not write reports, thus we might have underestimated this motivational item. More recently, some German medical schools introduced new requirements for international electives. Students now require language certificates to get credit for their elective. The language level B2 is now mandatory for many destinations. This high standard may have affected students’ choices who are now probably more likely to visit English-speaking countries as this is the first foreign language of many Germans.

Moreover, all reports were anonymous. While this may go along with advantages and disadvantages at the same time, we must emphasize that we could therefore not analyze students’ characteristics (such as demographic data, prior elective experience or the year of medical education). We believe that such information could have improved the presented study.

Finally, many reports were mere descriptions of an elective and did not include motivations for an abroad elective; approximately 33% of elective reports included a discussion on motivations and expectations, a low percentage of the overall number of reports. Nevertheless, we believe that our data may support medical schools, universities and other organizations to better understand the flow of students going to Africa and to tailor country-specific pre-departure trainings that may support students during their abroad stay.

## Conclusions

Abroad electives in Africa are popular among German-speaking medical students. Based on our findings, the most commonly reported destinations include the Republic of South Africa and Tanzania, where students often undertake electives in surgical disciplines. Elective motivations are diverse and while the majority of students go abroad to improve their clinical skills, a non**-**neglectable proportion of students in this sample expressed the motivation to “engage in operations and procedures not possible at home”, which raises many ethical issues, and calls for country- and context-specific pre-departure trainings to cover an often overlooked deontological aspect of medical training. Finally, our study has demonstrated a decrease in the number of overseas electives, most likely a consequence of the SARS-CoV-2 pandemic. We believe medical schools and universities should embrace this period to restructure their international elective, based on information provided from our data.

## Data Availability

All data associated with this paper will be made available upon reasonable request.

## Abbreviations

UNICEF: United Nations International Children's Emergency Fund
RSA: Republic of South Africa

## References

[1] Kumwenda B, Dowell J, Daniels K, Merrylees N (2015). Medical electives in sub-Saharan Africa: a host perspective. Med Educ.

[2] Ebrahimi-Fakhari D, Agrawal M, Wahlster L. International electives in the final year of German medical school education—a student’s perspective. GMS Z Med Ausbild [Internet]. 2014; 31(3). 10.3205/zma000918 PMC4152990 25228928

[3] Einterz EM (2008). The medical student elective in Africa: advice from the field. CMAJ.

[4] Storz MA, Lederer A-K, Heymann EP (2021). German-speaking medical students on international electives: an analysis of popular elective destinations and disciplines. Global Health.

[5] Storz M. Länder. In: PJ und Famulatur im Ausland. Springer-Lehrbuch. Springer, Berlin, Heidelberg. 2018. 10.1007/978-3-662-57657-1_7.

[6] Thompson MJ, Huntington MK, Hunt DD, Pinsky LE, Brodie JJ (2003). Educational effects of international health electives on U.S. and Canadian medical students and residents: a literature review. Acad Med.

[7] Cherniak WA, Drain PK, Brewer TF (2013). Educational objectives for international medical electives: a literature review. Acad Med.

[8] Fotheringham EM, Craig P, Tor E (2018). International medical electives in selected African countries: a phenomenological study on host experience. Int J Med Educ.

[9] Vlot JA, Blanter AI, Jonker EFF, Korse NS, Hack E, Visser LG (2020). Travel preparation and health risks in Dutch and Belgian medical students during an elective in low- or middle-income countries: a prospective self-reporting cohort study. Travel Med Infect Dis.

[10] Kironji AG, Cox JT, Edwardson J, Moran D, Aluri J, Carroll B (2018). Pre-departure training for healthcare students going Abroad: impact on preparedness. Ann Glob Health.

[11] Drain PK, Primack A, Hunt DD, Fawzi WW, Holmes KK, Gardner P (2007). Global health in medical education: a call for more training and opportunities. Acad Med.

[12] Hayashi M, Son D, Nanishi K, Eto M (2020). Long-term contribution of international electives for medical students to professional identity formation: a qualitative study. BMJ Open.

[13] Johnston N, Sandys N, Geoghegan R, O’Donovan D, Flaherty G. Protecting the health of medical students on international electives in low-resource settings. J Travel Med. 2018;25(1) 10.1093/jtm/tax092 29394388

[14] Tyagi S, Corbett S, Welfare M (2006). Safety on elective: a survey on safety advice and adverse events during electives. Clin Med (Lond).

[15] Adenrele AR (2017). Maintaining peace and security in Sub-Saharan Africa—the tragic connection between corruption, bad governance and criminality. Asian Res J Arts Soc Sci.

[16] Cilliers DJ (2003). Terrorism and Africa. Afr Secur Rev.

[17] Hope KR (2018). Police corruption and the security challenge in Kenya. Afr Secur.

[18] Banini DK, Powell J, Yekple M (2020). Peacekeeping as coup avoidance: lessons from Ghana. Afr Secur.

[19] Stoddard E (2019). Revolutionary Warfare? Assessing the character of competing factions within the Boko Haram insurgency. Afr Secur.

[20] Fenollar F, Mediannikov O (2018). Emerging infectious diseases in Africa in the 21st century. New Microbes and New Infections.

[21] Maeda JM, Nkengasong JN (2021). The puzzle of the COVID-19 pandemic in Africa. Science.

[22] Ramalho AR, Vieira-Marques PM, Magalhães-Alves C, Severo M, Ferreira MA, Falcão-Pires I (2020). Electives in the medical curriculum—an opportunity to achieve students’ satisfaction?. BMC Med Educ.

[23] Jolly B (2009). A missed opportunity. Med Educ.

[24] Chenot J-F. Undergraduate medical education in Germany. Ger Med Sci. 2009;7:Doc02. 10.3205/000061 PMC2716556 19675742

[25] Storz M. Famulatur. In: PJ und Famulatur im Ausland. Springer-Lehrbuch. Springer, Berlin, Heidelberg. 2018. 10.1007/978-3-662-57657-1_2.

[26] Zavlin D, Jubbal KT, Noé JG, Gansbacher B. A comparison of medical education in Germany and the United States: from applying to medical school to the beginnings of residency. Ger Med Sci. 2017;15. 10.3205/000256 PMC5617919 29051721

[27] Nikendei C, Weyrich P, Jünger J, Schrauth M (2009). Medical education in Germany. Med Teach.

[28] Egiz A, Storz MA (2021). The COVID-19 pandemic: doom to international medical electives? Results from two German elective databases. BMC Res Notes.

[29] About Africa [Internet]. UNDP in Africa. [cited 2021 Apr 6]. Available from: https://www.africa.undp.org/content/rba/en/home/regioninfo.html.

[30] Daniels K, Thomson E, Nawagi F, Flinkenflögel M (2020). Value and feasibility of South-South Medical Elective Exchanges in Africa. BMC Med Educ.

[31] Lumb A, Murdoch-Eaton D. Electives in undergraduate medical education: AMEE Guide No. 88. Med Teach. 2014;36(7):557–72. 10.3109/0142159X.2014.907887 24787526

[32] Chao FY. International medical electives: time for a rethink? [Internet]. Australian Medical Student J. [cited 2021 Apr 8]. Available from: https://www.amsj.org/archives/3381.

[33] Elit L, Hunt M, Redwood-Campbell L, Ranford J, Adelson N, Schwartz L (2011). Ethical issues encountered by medical students during international health electives. Med Educ.

[34] Wiskin C, Dowell J, Hale C (2018). Beyond ‘health and safety’—the challenges facing students asked to work outside of their comfort, qualification level or expertise on medical elective placement. BMC Med Ethics.

[35] Banatvala N, Doyal L (1998). Knowing when to say “no” on the student elective. BMJ.

[36] Petrosoniak A, McCarthy A, Varpio L (2010). International health electives: thematic results of student and professional interviews. Med Educ.

[37] Hanson L, Harms S, Plamondon K (2011). Undergraduate international medical electives: some ethical and pedagogical considerations. J Stud Int Educ.

[38] Crump JA, Sugarman J (2008). Ethical considerations for short-term experiences by trainees in global health. JAMA.

[39] Banerjee A (2010). Medical electives: a chance for international health. J R Soc Med.

[40] Huish R. The ethical conundrum of international health electives in medical education. J Global Citizenship Equity Educ [Internet]. 2012;2(1).

[41] Choi B, Jegatheeswaran L, Minocha A, Alhilani M, Nakhoul M, Mutengesa E (2020). The impact of the COVID-19 pandemic on final year medical students in the United Kingdom: a national survey. BMC Med Educ.

[42] McMaster D, Veremu M, Jonas KM. Should international medical electives to resource-poor countries continue during COVID-19? J Travel Med. 2020;27(6). 10.1093/jtm/taaa071 PMC7239176 32374832

[43] Watson DA, Cooling N, Woolley IJ (2019). Healthy, safe and effective international medical student electives: a systematic review and recommendations for program coordinators. Trop Dis, Travel Med Vaccines.

[44] CDC Global Health—South Africa—CDC’s HIV/AIDS Care and Treatment Programs: TB and HIV [Internet]. 2019 [cited 2021 Apr 8]. Available from: https://www.cdc.gov/globalhealth/countries/southafrica/what/tb_hiv.htm.

[45] Johnston N, Sandys N, Geoghegan R, O’Donovan D, Flaherty G (2018). Protecting the health of medical students on international electives in low-resource settings. J Travel Med.

[46] Dao TL, Gautret P (2021). Patterns of diseases in health students abroad: a systematic review. Travel Med Infect Dis.

